# Insulin Sensitivity Is Not Decreased in Adult Patients With Hypopituitarism Without Growth Hormone Replacement

**DOI:** 10.3389/fendo.2019.00534

**Published:** 2019-08-07

**Authors:** Alejandro Rosell Castillo, Aglecio Luiz de Souza, Sarah Monte Alegre, Yeelen Ballesteros Atala, Denise Engelbrecht Zantut-Wittmann, Heraldo Mendes Garmes

**Affiliations:** ^1^Endocrinology Division, Department of Clinical Medicine, Faculty of Medical Sciences, University of Campinas, Campinas, Brazil; ^2^Department of Internal Medicine, Faculty of Medical Sciences, State University of Campinas, Campinas, Brazil

**Keywords:** hypopituitarism, GH deficiency, euglycemic hyperinsulinemic clamp, insulin sensitivity, fat mass, free fat mass

## Abstract

Decreased insulin sensitivity in patients with hypopituitarism without GH replacement (pHP-WGHR) remains conflicting in literature. It is known that these patients present a decrease in free fat mass and an increase in fat mass. Typically, these kinds of alterations in body composition are associated with a decrease in insulin sensitivity; however, there is no consensus if this association is found in pHP-WGHR. Thus, we investigated pHP-WGHR regarding insulin sensitivity by euglycemic hyperinsulinemic clamp, the gold standard method, and body composition. In a cross-sectional study, we evaluated 15 pHP-WGHR followed up in a Service of Neuroendocrinology and 15 individuals with normal pituitary function as a control group with similar age, gender and body mass index. Insulin sensitivity was evaluated by euglycemic hyperinsulinemic clamp and homeostatic model assessment insulin resistance (HOMA-IR). *Kappa* coefficient evaluated the agreement between these two methods. Percentage of fat mass, percentage of free fat mass, fat mass weight and free fat mass weight were assessed by electrical bioimpedance. The pHP-WGHR presented similar insulin sensitivity to control group by euglycemic hyperinsulinemic clamp, both by the M-value, (*p* = 0.0913) and by the area under the glucose infusion rate curve, (*p* = 0.0628). These patients showed lower levels of fasting glycemia (*p* = 0.0128), insulin (*p* = 0.0007), HOMA-IR (*p* = 0.009). HOMA-IR shows poor concordance with euglycemic hyperinsulinemic clamp (Kappa = 0.16) in pHP-WGHR, while in the control group the agreement was good (Kappa = 0.53). The pHP-WGHR presented higher values of percentage of fat mass (*p* = 0.0381) and lower values of percentage of free fat mass (*p* = 0.0464) and free fat mass weight (0.0421) than the control group. This study demonstrated that the insulin sensitivity evaluated by euglycemic hyperinsulinemic clamp in pHP-WGHR was similar to individuals with normal pituitary function, despite the pHP-WGHR presenting higher fat mass percentage. HOMA-IR was not a good method for assessing insulin sensitivity in pHP-WGHR.

## Introduction

Hypopituitarism (HP) results from complete or partial deficiency in pituitary hormones from several etiologies and includes adrenal insufficiency, hypothyroidism, hypogonadism, growth hormone deficiency (GHD) and rarely diabetes insipidus (1). These patients need a replacement of these hormones according to current guidelines standard (1–3).

Decreased in insulin sensitivity has been noted in patients with hypopituitarism without GH replacement (pHP-WGHR), an alteration attributed to the GHD (4–7). However, there are animals and human studies suggesting that insulin sensitivity is not decreased in GHD (7–12). It is known that these patients present a decrease in free fat mass (FFM) and an increase in fat mass (FM) (13–16). Classically, these kind of alterations in body composition are associated with a decrease in insulin sensitivity, albeit there is no consensus if this association is found in pHP-WGHR. On the other hand, some authors have demonstrated the opposite; increased insulin sensitivity in these patients when compared with a control group matched for age, sex and body mass index (BMI) (9–12). We highlight that the authors of the mentioned studies that evaluated insulin sensitivity used different methods of evaluation to the homeostatic model assessment (HOMA-IR), the frequently sampled intravenous glucose tolerance tests with minimal model analysis or euglycemic hyperinsulinemic clamp (EHC) (4–12).

Due to these contradictory results and scarce studies with the gold standard method to evaluate insulin sensitivity, euglycemic hyperinsulinemic clamp, we performed a study using this method in patients with hypopituitarism without growth hormone replacement compared to a control group composed by individuals with normal pituitary function paired by BMI, gender, and age and additionally, we evaluated the characteristics of body composition in these groups.

## Materials and Methods

### Study Design

This was a cross-sectional study. We evaluated patients with a previous diagnosis of HP; all followed in the Service of Neuroendocrinology of the hospital of the University of Campinas between the years 2016 and 2018. All patients were on levothyroxine, prednisone, estrogen and progesterone or testosterone replacement and none on GH therapy. We evaluated the age, gender and BMI clinically, etiology of HP, basal metabolic rate (BMR), BC variables such as percentage of fat mass (PFM), percentage of free fat mass (PFFM), fat mass weight (FMW), and free fat mass weight (FFMW) by means of electrical bioimpedance. Besides that, we evaluated fasting glucose and insulin used for calculated HOMA-IR and insulin sensitivity by Euglycemic hyperinsulinemic clamp. The associations between body composition characteristics and insulin sensitivity of HP patients were compared with the control subjects. The control group consisted of individuals with normal pituitary function, paired by age, gender and BMI. All patients and control subjects included in this study were of Caucasoid etnia. The local Ethics Committee approved the study (CAAAE 1.531.415) and all participants written and signed an informed consent form.

### Patients

Fifteen patients with HP were included without residual tumor or previous functioning tumor between 20 and 59 years old. Data regarding age, sex, height, and weight measures were assessed. The diagnosis of HP was made in the presence of deficiency of thyroid-stimulating hormone (TSH), free T4 (FT4), adrenocorticotropic hormone (ACTH), basal cortisol, growth hormone (GH), insulin-like growth factor (IGF-1), luteinizing hormone (LH), follicle stimulating hormone (FSH), total and free testosterone (in male patients) or estradiol (in women). An insulin tolerance test was performed for the diagnosis of GH and cortisol deficiency. All patients were replaced with levothyroxine (mean dose 1.56 mcg/kg/day), prednisone (mean dose 2.2 mg/m^2^/day) and estrogen/progesterone (17β estradiol 2 mg + norethisterone acetate 1 mg/day) or testosterone (testosterone cypionate 200 mg every 3 weeks), dosages were monitored and adjusted as required according to current guideline standard. Adequate replacement was assumed when medication had not been adjusted for at least 6 months, patients had no complaints, and basal hormone levels were within the recommended values. Patients participating in the study do not present contraindications for GH replacement.

Exclusion criteria were residual pituitary tumor or previous functioning tumor, glucose intolerance, diabetes mellitus, acutely infirm patients, malignant tumors, active inflammatory disease, class III/IV heart failure (NYHA classification), severe hepatic disease (low albumin or increased IRN), advanced kidney disease (stage 4 or 5), HIV or psychiatric diseases.

### Control Group

Fifteen individuals without pituitary disease with normal pituitary function defined by previous hormonal evaluation, paired by age, gender and BMI, were recruited as a control group in the same period and following the same criteria for exclusion applied to patients with HP. The individuals were recruited among the relatives of patients from the outpatient neuroendocrinology clinic. The same clinical, insulin sensitivity and body composition characteristics evaluated in the group of patients with HP were assessed in the control group.

### Clinical and Body Composition Parameters

Clinical data were recorded consisting of age, sex, BMI, and etiology of HP. BMI was calculated based on the ratio between body mass (in kg) and squared height (in meters). In all the study subjects, BMR and BC variables were evaluated by electrical bioimpedance with a Biodynamics monitor (Biodynamics Corp., Seattle, WA, USA) as described by Boulier et al. (17). The measurements were taken in the morning after each subject had fasted at least 10 h and bladder voided. The subject had been supine for at least 5 min, arms relaxed at the sides but not touching the body and thighs separated.

### Laboratory Measurements

Insulin was measured by Human Insulin Elisa Technique (Millipore, Billerica, Massachusetts, USA), RV: 2.0–200 μU/ml (CV = 4.64%) and plasma glucose was measured with the glucose oxidase method using an YSI glucose analyzer (YSI 2300-Stat Plus analyzer; YSI, Yellow Springs, OH, USA; RV: 70–100 mg/dl). HOMA-IR was calculated by the formula insulin × glucose/22.5 (18, 19). Individuals who presented HOMA-IR > 2.7) were considered insulin resistant (20).

### Clamp Study

The Euglycemic hyperinsulinemic clamp study, which was carried out after an overnight (12 to 14 h) fast, consisted of 2 h of euglycemic insulin infusion at a rate of 40 μU/min per meter squared of body surface area and was preceded by a 2-h control period as previously described (21). A polyethylene, 20-gauge catheter was inserted into an antecubital vein for the infusion of insulin and glucose. Another catheter was inserted retrograde into a wrist vein, and the hand was placed in a heated box (50–60°C) for the sampling of arterialized blood. Following this procedure, the patients rested for at least 30 min in the supine position. The infusion was adjusted according to glucose determinations made every 5 min on a glucose analyzer (Yellow Springs Instruments, Yellow Springs, OH). The venous samples for insulin measurements were obtained at 30 min intervals during the 2 h of the clamp. For calculation of insulin sensitivity from the Glucose Infusion Rate (GIR) curve and the glucose disposal rate (M-value) (milligrams per kilogram per minute) were calculated from the infusion rate of exogenous glucose during the second hour of the insulin clamp period, M-value and GIR were normalized per kg FFM and per kg FFM/Glycemia, this last normalization was multiplied by 100 (21, 22). At the end, subjects who presented M <4.8 mg (corrected by MM) were considered insulin resistant (23).

### Statistical Analysis

To describe the profile of the sample according to the variables under study, the frequency tables of the categorical variables with absolute (*n*) and percentage (%) values were used. Descriptive statistics of the numerical variables, with values of mean, standard deviation, minimum values and maximum and median were performed. Categorical variables were compared using the Chi-square test and, when necessary, Fisher exact test. Mann-Whitney test was used to compare numerical variables. To evaluate the agreement between the measurement M-value (corrected by FFM) and the HOMA-IR, the Kappa test was used (24). Confidence interval (CI) was 95%. The level of significance adopted for the study was 5%.

## Results

### Clinical Characteristics of Hypopituitary Patients

The etiology of HP included congenital pituitary hypoplasia (6 patients), non-functioning macroadenomas (postsurgical-3 patients), craniopharyngioma (postsurgical-3 patients), empty sella (2 patients) and Sheehan's Syndrome (1 patient). The deficiencies of pituitary hormones and treatment of HP patients compared to control group are presented in Table 1. The individual description of each patient in the study group regarding treatment, levels of IGF-1, insulin sensitivity and body composition are shown in Table 2.

**Table 1:** Frequency of pituitary hormone deficiencies and replacement therapy in patients with HP and control subjects.

**Variable**	**Patients with HP** **(*n* = 15)**	**Control subjects** **(*n* = 15)**
GH deficiency/treatment	15/0	0/0
ACTH deficiency/treatment	15/15	0/0
TSH deficiency/treatment	15/15	0/0
LH-FSH deficiency/treatment	15/14	0/0
ADH deficiency/treatment	4/4	0/0

*HP, Hypopituitarism*.

**Table 2:** Clinical characteristics, replacement therapy, insulin sensitivity and body composition of patients with hypopituitarism.

**pHP-WGHR (*n* = 15)**	**Sex**	**Age (years)**	**BMI (kg/m^2^)**	**Treatment**	**IGF-1 (ng/ml)**	**HOMA-IR**	**M/FFM (mg/kg/min)**	**M/FFM/Gly (mg/kg/min)**	**PFFM (%)**	**PFM (%)**
Pt-1	F	32	22.06	P-L-Es/Pg	22.67	0,3	6	8.51	65.4	34.6
Pt-2	M	50	31.92	P-L-T	18.8	3.1	4.94	5.90	65.7	34.3
Pt-3	F	56	41.21	P-L	47.64	1.6	2.81	2.96	56.6	43.4
Pt-4	M	50	17.78	P-L-T	31	0.4	6.83	9.36	76.2	23.8
Pt-5	M	41	31.50	P-L-T-D	30.11	1.7	2.87	3.11	63.5	36.5
Pt-6	F	42	46.00	P-L-Es/Pg	31.5	0.4	4.45	5.83	66.3	33.7
Pt-7	M	40	18.80	P-L-T	34.44	0.3	10.52	15.02	75.9	24.1
Pt-8	M	32	24.61	P-L-T-De	52.6	0.4	8.78	11.61	77.5	22.5
Pt-9	M	23	21.90	P-L-T	40.1	0.4	10.63	13.57	62.2	37.8
Pt-10	M	24	17.70	P-L-T-D	18.39	0.4	13.7	19.96	65.4	34.6
Pt-11	M	25	20.28	P-L-T	36.33	0.4	12.93	16.82	79.6	20.4
Pt-12	M	22	35.08	P-L-T-D	69.65	3.3	3.02	3.58	68.5	31.5
Pt-13	F	24	21.52	P-L-Es/Pg	25.4	0.4	12.53	16.36	70.3	29.7
Pt-14	F	22	23.14	P-L-Es/Pg	36.69	0.3	4.84	6.5	70.1	29.9
Pt-15	F	22	19.80	P-L-Es/Pg	71.68	0.4	7.84	8.82	67.8	32.2

*pHP-WGHR, patients with hypopituitarism without GH replacement; Pt, patient; P, prednisone; L, levothyroxine; Es/Pg, Estrogen/Progesterone; D, Desmopressin; HOMA-IR, homeostatic model assessment of insulin resistance; M/FFM, M-value normalized per kg Free Fat Mass; M/FFM/Gly, M-value normalized per kg Free Fat Mass and Glycemia; PFM, Percentage of Fat Mass; PFFM, Percentage of Free Fat Mass*.

Our study included 15 patients with hypopituitarism (6 females) and a control group of 15 individuals (6 females). The two groups did not present significant differences in age, gender and BMI. On the other hand, levels of IGF-1 were significantly lower in patients with HP: Table 3.

**Table 3:** Comparative analysis of clinical characteristics, body composition and insulin sensitivity between patients with HP and control subjects.

**Variable**	**Patients with HP** **(*n =* 15)**	**Control subjects** **(*n* = 15)**	***p*-value**
Sex (female)	6 (40%)	6 (40%)	1.000
Age (years) Mean ± SD	33.7 ± 11.9	34.0 ± 11.4	0.8679
Median (min-max)	32.0 (22.0-56.0)	30.0 (21.0–54.0)	
BMI (kg/m^2^) Mean ± SD	26.2 ± 8.9	27.8 ± 8.8	0.2808
Median (min-max)	22.1 (17.7–46.0)	23.6 (17.9–48.6)	
IGF-1(ng/ml) Mean ± SD	38.1 ± 16.1	170.9 ± 67.9	<.0001
Median (min-max)	34.4 (18.4–71.7)	157.2 (75.4–286.1)	
F-Gly (mg/dl) Mean ± SD	78.3 ± 7.6	86.0 ± 7.8	0.0128
Median (min-max)	77.5 (69.7–92.5)	87.2 (73.2–97.4)	
F-Insul (μU/ml) Mean ± SD	3.4 ± 3.7	8.5 ± 7.6	0.0007
Median (min-max)	2.0 (0.9–14.9)	4.9 (2.1–28.0)	
HOMA-IR Mean ± SD	0.9 ± 1.0	1.8 ± 1.7	0.0090
Median (min-max)	0.4 (0.3–3.3)	0.9 (0.4–6.2)	
M/FFM (mg/kg/min) Mean ± SD	7.5 ± 3.8	5.5 ± 2.1	0.0913
Median (min-max)	6.8 (2.8–13.7)	5.7 (2.4–9.5)	
M/FFM/Gly (mg/kg/min) Mean ± SD	9.9 ± 5.4	6.4 ± 2.7	0.0890
Median (min-max)	8.8 ± (2.9–19.9)	6.1 (2.5–11.2)	
PFFM (%) Mean ± SD	68.7 ± 6.3	75.0 ± 9.8	0.0464
Median (min-max)	67.8 (56.6–79.6)	75.3 (55.6–92.9)	
PFM (%) Mean ± SD	31.3 ± 6.3	25.0 ± 10.2	0.0381
Median (min-max)	32.2 (20.4–43.4)	4.7 (7.1–44.4)	
BMR (cal/day) Mean ± SD	1500.4 ± 494.6	1837.8 ± 459.4	0.0421
Median (min-max)	1343.0 (926.0–2525.0)	1893.0 (1181.0–2829.0)	

*Values are shown as mean (standard deviation), Median (min-max) or number and frequency (percentage)*.

*HP, Hypopituitarism; BMI, body mass index; F-insulin, Fasting insulin; F-Glycemia, Fasting Glycemia; HOMA-IR, homeostatic model assessment of insulin resistance; M/FFM, M-value normalized per kg Free Fat Mass; M/FFM/Gly, M-value normalized per kg Free Fat Mass and Glycemia; PFM, Percentage of Fat Mass; PFFM, Percentage of Free Fat Mass; BMR, Basal Metabolic Rate*.

### Insulin Sensitivity and Glucose Metabolism in pHP-WGHR

**Figure 1:**
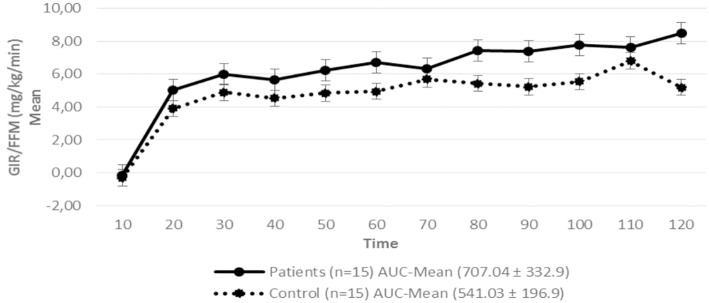
Glucose Infusion Rate (GIR) corrected for the Free Fat Mass (FFM) in patients with hypopituitarism and control subjects during the Euglycemic Hyperinsulinemic Clamp (mean values every 10 min) *p* = 0.16. AUC, Area Under the Curve. For the calculation of the areas under the curve (AUC) only the average values of the last hour were used.

**Figure 2:**
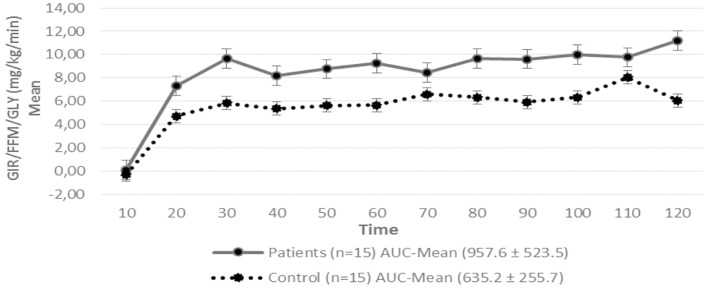
Glucose Infusion Rate (GIR) corrected for the Free Fat Mass (FFM) and Glycemia (GLY) in patients with hypopituitarism and control subjects during the Euglycemic Hyperinsulinemic Clamp (mean values every 10 min) *p* = 0.12. AUC, Area Under the Curve. For the calculation of the areas under the curve (AUC) only the average values of the last hour were used.

**Figure 3:**
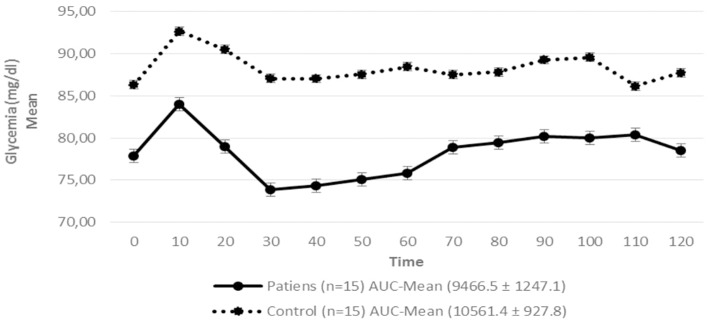
Serum glycemia in patients with hypopituitarism and control subjects during the Euglycemic Hyperinsulinemic Clamp (mean values from time 0, every 10 min) *p* = 0.009. AUC, Area Under the Curve. For the calculation of the areas under the curve (AUC) mean values of glycemia during the clamp were used.

**Figure 4:**
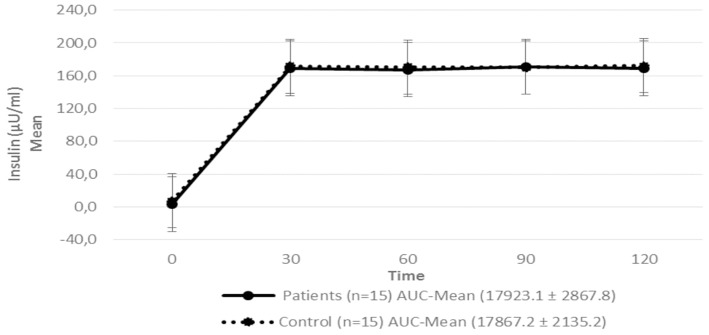
Serum insulin level in patients with hypopituitarism and control subjects during the Euglycemic Hyperinsulinemic Clamp (mean: 0, 30, 60, 90, and 120 min) *p* = 0.7089. AUC, Area Under the Curve. For the calculation of the areas under the curve (AUC) mean values of insulin during the clamp were used.

Regarding insulin sensitivity, we found that M-value and area under the GIR curve were not statistically different between patients and control group as shown in Table 3, Figures 1, 2 respectively. Patients with hypopituitarism without GH replacement showed significant lower levels of fasting glycemia, insulin and HOMA-IR (Table 3). We found that the glycemia curve values during the clamp were significantly lower in the group of pHP-WGHR compared with control subjects (Figure 3). There were no differences between the insulin levels in pHP-WGHR patients and control subjects (Figure 4). In the assessment of SI, the agreement of the HOMA-IR with the EHC method in the pHP-WGHR was weak (Kappa = 0.16; CI95% = 0.000–0.734) and in control subjects was moderate (Kappa = 0.53; CI95% = 0.062–0.990).

### Body Composition of Patients With HP-WGHR

Patients with HP-WGHR had higher percentage of fat mass and lower percentage of free fat mass and basal metabolic rate, compared with control subjects (Table 3).

## Discussion

Our study demonstrated that, using the EHC method, pHP-WGHR presented similar insulin sensitivity when compared to the control group as lower levels of fasting glycemia, fasting insulin and HOMA-IR. Additionally, we verified that HOMA-IR had a poor concordance with the EHC method for assessing insulin sensitivity in pHP-WGHR. At the same time, we observed that pHP-WGHR had higher fat mass and lower amount of free fat mass.

Our findings are not in agreement to some similar studies in the literature that showed decreased insulin sensitivity in pHP-WGHR (5, 6). Besides the difference between the studied populations, this difference could be explained by the management of hypopituitarism, because currently the recommended hormone replacement doses are more physiological than those used previously (1).

We also found differences in other markers associated to insulin sensitivity, such as lower levels of fasting glycemia, insulin and HOMA-IR, in accordance with other authors and suggesting that pHP-WGHR do not have decreased insulin sensitivity (8–12, 25–29).

Interestingly, the presence of lower blood glucose levels in the patients during the clamp, despite similar insulin levels when compared to the control group, may be a consequence of a greater sensitivity of peripheral tissues to exogenous insulin (30). Another explanation would be the decrease of glycogen stores in muscle and liver, as already demonstrated in animal studies with GH receptor knockout (31, 32). This finding could explain why patients with hypopituitarism have a higher hypoglycemic response to insulin and take longer to recover from hypoglycemia (30).

The deficiency of GH (insulin counter-regulatory hormone) and decrease of the glucose hepatic production rate, may explain the lower glucose levels in these patients (30, 33, 34). Furthermore, the decrease of serum IGF-1 in these patients would lead to an increased expression of the IGF-1 receptor. The binding of insulin to IGF-1 receptors could justify the lower blood insulin levels found in our patients. Additionally, the action of insulin via the IGF-1 receptor could decrease glucose levels, as previously demonstrated in animal models (35), which could result in lower values of HOMA-IR in these patients.

It is important to highlight that these patients presented an increased insulin sensitivity by the HOMA-IR method, but not by the clamp, the gold standard method. This difference could be explained by the different concepts of two methods. Thus, HOMA-IR evaluate insulin sensitivity in the fasting state, since patients with GHD presents a decrease in the rate of liver glucose production, fundamentally in fasting, which would lead to lower levels of glycemia and insulin, resulting in lower HOMA-IR values (30, 33). On the other hand, the hyperinsulinemic euglycemic clamp is based on the concept that under constant glycemic and hyperinsulinemic conditions, with suppression of liver glucose production, the amount of glucose consumed by the tissues should be equivalent to the amount of glucose infused during the test (36). This suppression of the hepatic glucose production would exclude a possible interference derived from GHD. So, it is possible to explain why the concordance index between HOMA-IR and clamp was poor in pHP-WGHR, suggesting that HOMA-IR could not be a good method for assessing insulin sensitivity in these patients, because this method is not able to define the influence of the liver or peripheral tissues on insulin sensitivity and does not take into account the decrease in the hepatic production of glucose presented in pPH-WGHR (36).

Patients with secondary adrenal insufficiency may frequently have residual cortisol secretion and need the optimization of the doses of glucocorticoids to avoid the consequences of excessive doses (37). Because of this, we performed the tailoring of glucocorticoids replacement in our patients. Few studies have evaluated whether glucocorticoid dosing and regimen change insulin sensitivity. In relation to the doses, a recent study has shown that doses ≤20 mg/day of hydrocortisone, which corresponds to 25 mg of cortisone acetate or 5 mg of prednisolone, does not alter insulin sensitivity when compared to 30 mg/day of hydrocortisone (38). Regarding the therapeutic regimen, a study performed with euglycemic hyperinsulinemic clamp showed that insulin sensitivity does not change when compared to traditional oral replacement with the continuous hydrocortisone infusion scheme mimicking the circadian rhythm (39).

As expected, our patients showed higher values of PFM and lower values of PFFM and BMR, in agreement with other studies, pointing to the classic characteristics of pHP-WGHR (13–16, 40–49), even with normal BMI (15). These findings could be the consequence of the anabolic effect of GH on muscle and lipolytic effect on adipose tissue (50).

It is known that increased percentage of fat mass in healthy individuals is directly related to decreased insulin sensitivity. Interestingly, the clamp method showed that, despite unfavorable body composition, pHP-WGHR did not present a decrease in insulin sensitivity when compared to healthy individuals with similar BMI, as has already been demonstrated in some experimental studies on mice (40–42). Similar results were also shown in the studies of a population of Ecuador patients with GH insensitivity due to a mutant GH receptor (Laron Syndrome) and in the cohort of patients from Itabaianinha (Brazil) who presented GHD due to a mutation of the GH releasing hormone receptor. We emphasize that in both populations, the HOMA-IR and quantitative insulin check index methods were used for the evaluation of insulin sensitivity (12, 13, 43, 44). One hypothesis to justify this finding is it may be due to a greater increase in subcutaneous than to visceral fat, as already demonstrated in mice with GH receptor knockout (40). The increased visceral fat is associated with decreased insulin sensitivity as opposed to subcutaneous fat (51, 52). However, it is important to emphasize that this method of electrical bioimpedanciometry could not distinguish these fat distributions.

Clinically, patients with increased fat mass have worsened insulin sensitivity, however we have shown that our pHP-WGHR do not present decreased insulin sensitivity despite unfavorable body composition. We also point out that additional studies are needed in pHP-WGHR with GH replacement therapy to understand the effects of GH on these parameters.

A limitation of this study was the relatively small sample size, due to the inclusion of patients with complete hypopituitarism, with no residual tumor, no previous functioning pituitary tumor and due to complex methodology of clamp study. Other studies using EHC had similar number of patients. Another limitation was the use of electrical bioimpedanciometry as a method for the evaluation of body composition. However, electric bioimpedanciometry has a good correlation with DEXA (53), the gold standard method for the body composition evaluation.

The main strength of our study was the use of the gold standard method in the evaluation of insulin sensitivity, the inclusion of patients with complete pituitary hormone deficiency and the presence of a similar control group in age, gender and BMI, since these characteristics exert great influence on the hormonal and metabolic variables studied (54).

## Conclusion

Our study verified that insulin sensitivity evaluated by euglycemic hyperinsulinemic clamp in pHP-WGHR had similar results in control subjects with normal pituitary function paired by age, gender and BMI, suggesting that the worsening of insulin sensitivity was not a characteristic of these patients, despite higher fat mass and lower free fat mass. HOMA-IR was not a good marker for assessing insulin sensitivity in pHP-WGHR. However, we believe that more studies are needed to better understand the effect of GH on insulin sensitivity and on the distribution of body fat.

## Data Availability

All datasets generated for this study are included in the manuscript/supplementary files.

## Author Contributions

AC patient selection, study design, literature search, data collection, data analysis, data interpretation, writing. AdS and SA data collection, data analysis, data interpretation. YA data analysis, data interpretation, writing. DZ-W study design, data analysis, data interpretation, and writing. HG patient selection, study design, literature search, data analysis, data interpretation, writing.

### Conflict of Interest Statement

The authors declare that the research was conducted in the absence of any commercial or financial relationships that could be construed as a potential conflict of interest.
